# Evaluation of a simple, rapid and field-adapted diagnostic assay for enterotoxigenic *E*. *coli* and *Shigella*

**DOI:** 10.1371/journal.pntd.0010192

**Published:** 2022-02-07

**Authors:** Sean Connor, Mirza Velagic, Xueyan Zhang, Fatema-Tuz Johura, Goutam Chowdhury, Asish K. Mukhopadhyay, Shanta Dutta, Munirul Alam, David A. Sack, Thomas F. Wierzba, Subhra Chakraborty

**Affiliations:** 1 Department of International Health, Johns Hopkins Bloomberg School of Public Health, Baltimore, Maryland, United States of America; 2 icddr,b, Formerly International Centre for Diarrhoeal Disease Research, Bangladesh, Dhaka, Bangladesh; 3 ICMR-National Institute of Cholera and Enteric Diseases, Kolkata, India; 4 Wake Forest School of Medicine, Winston Salem, North Carolina, United States of America; Creighton University, UNITED STATES

## Abstract

Understanding the global burden of enterotoxigenic *E*. *coli* (ETEC) and Shigella diarrhea as well as estimating the cost effectiveness of vaccines to control these two significant pathogens have been hindered by the lack of a diagnostic test that is rapid, simple, sensitive, and can be applied to the endemic countries. We previously developed a simple and rapid assay, Rapid Loop mediated isothermal amplification based Diagnostic Test (RLDT) for the detection of ETEC and *Shigella spp*. (Shigella). In this study, the RLDT assay was evaluated in comparison with quantitative PCR (qPCR), culture and conventional PCR for the detection of ETEC and Shigella. This validation was performed using previously collected stool samples from endemic countries, from the travelers to the endemic countries, as well as samples from a controlled human infection model study of ETEC. The performance of RLDT from dried stool spots was also validated. RLDT resulted in excellent sensitivity and specificity compared to qPCR (99% and 99.2% respectively) ranging from 92.3 to 100% for the individual toxin genes of ETEC and 100% for Shigella. Culture was less sensitive compared to RLDT. No significant differences were noted in the performance of RLDT using samples from various sources or stool samples from moderate to severe diarrhea or asymptomatic infections. RLDT performed equally well in detection of ETEC and Shigella from the dried stool samples on filter papers. This study established that RLDT is sufficiently sensitive and specific to be used as a simple and rapid diagnostic assay to detect ETEC and Shigella in endemic countries to determine disease burden of these pathogens in the national and subnational levels. This information will be important to guide public health and policy makers to prioritize resources for accelerating the development and introduction of effective preventative and/or treatment interventions against these enteric infections.

## Author summary

Enterotoxigenic *E*. *coli* (ETEC) and *Shigella spp* (Shigella) causes significant global morbidity and mortality, especially in low-and middle-income countries (LMICs). Since culture methods to detect Shigella are not sensitive, and the methods used to detect ETEC have not been feasible outside of specialized, well-equipped laboratories, the true burden of these pathogens at national and sub-national levels are mostly not available. Morbidity and mortality estimates, for these two pathogens are crucial to assess their relative public health importance in LMICs. We developed a simple and rapid diagnostic assay called the RLDT (Rapid Loop-mediated isothermal amplification based Diagnostic Test) for detection of ETEC and Shigella. In this study we evaluated RLDT compared to other currently available assays using previously collected stool samples. Our data showed that the RLDT assay exhibited high sensitivity and specificity for detection of ETEC and Shigella, with its result available within 50 minutes. The sensitivity of RLDT was higher than culture for these pathogens. We conclude that RLDT could be used as a rapid and simple diagnostic test to determine the burden of ETEC and Shigella in LMICs as well as in clinical vaccine trials of these pathogens.

## Introduction

Enterotoxigenic *Escherichia coli* (ETEC) and *Shigella spp*. (Shigella) remain among the most common bacterial causes of diarrhea-associated morbidity and mortality in the children living in the low-and middle-income countries (LMIC) [1–2] as well as in the travelers and military personnel from the high-income countries [3].

In spite of the high impact of these enteric pathogens on child health [4,5,6] a simple, sensitive and specific diagnostic test for ETEC and Shigella is lacking. Standard culture methods for Shigella lack sensitivity and assays for ETEC are only available in well-equipped research laboratories and therefore, the estimates of the burden of these pathogens are not readily available from the LMICs [1,6,7]. Consequently, regional public health officials and policy makers lack a clear appreciation of the impact that ETEC and Shigella have on child and adult health in their countries. The paucity of data, particularly at the national and sub-national levels, has created uncertainties in the reported mortality estimates [8,9]. Morbidity and mortality estimates are critical to support investment decision making, and ultimately policy recommendations for vaccine development and use [9]. As promising vaccine candidates for ETEC and Shigella move toward field trials in endemic areas, an improved understanding of the epidemiology of both pathogens and identification of high transmission hotspots of these diseases will be critical to plan Phase III trials to assess the potential benefits of vaccine use [8]. In this regard, a sensitive, simple and rapid diagnostic method which can be applied in the resource poor settings, where these diseases are endemic is essential. One may draw an analogy with rotavirus where a simple diagnostic test allowed many countries to assess their disease burden and determine the effectiveness of vaccine introduction making it possible to accelerate introduction of the vaccine in the critically required countries in LMICs [10,11]. Such a simple, rapid and as well as sensitive diagnostic assay which can be applied in resource poor endemic settings with limited laboratory infrastructure is currently lacking for ETEC and Shigella. [1,7,12].

The need for such an assay, led us to the development of the Rapid LAMP (Loop-mediated Isothermal Amplification) based Diagnostic Test (RLDT) for ETEC and Shigella [13]. RLDT is a simple test which detects ETEC and Shigella directly from stool in 50 minutes.

In this study, we evaluated RLDT at the Johns Hopkins University (JHU) using previously collected stool samples in comparison with quantitative PCR (qPCR), conventional PCR and available culture data for the detection of ETEC and Shigella. Our primary aim was to determine if RLDT could be applied to detect ETEC and Shigella in stool samples collected from various types of population and endemic settings. These included a) stool samples collected from the diarrhea patients in India and Bangladesh; b) stool samples from the travelers from high income countries traveling to Guatemala and Mexico c) stool samples from a controlled human infection model (CHIM) study on ETEC with healthy American volunteers. We validated performance of RLDT using stool from cases of moderate to severe diarrhea (MSD) as well as in samples from asymptomatic individuals. In addition, we evaluated the performance of RLDT from dried stool samples on filter paper.

## Materials and methods

Ethics Statement: The above mentioned studies were approved by, the Ethical Review Committee of the icddr,b the Ethical review committee of the NICED and the Institutional Review Board of Johns Hopkins School of Public Health; IRB 00002722.

To evaluate RLDT, stored stool samples from the following studies were included.

### Study 1: ETEC surveillance study in Bangladesh

A surveillance study for ETEC diarrhea was conducted in Upazila Health Complex of Mathbaria and Chhatak, Bangladesh in a collaboration between JHU and the icddr,b during 2014–2016. Patients with acute watery diarrhea presenting in health facilities, were enrolled in this study [14]. After obtaining informed consent from the patients, stool samples were collected and were cultured on MacConkey agar and five lactose fermenting *E*. *coli* like colonies were selected and screened for detection of ETEC targeting the toxin genes LT, STh and STp in a multiplex conventional PCR [15].

Two hundred and sixty-one stored stool samples and their culture data were included in the current study.

### Study 2: Traveler’s study

This was a prospective, double-blind, randomized, placebo-controlled trial of a killed oral ETEC vaccine in persons travelling from the United States to Antigua, Guatemala or Cuernavaca, Mexico during 1998 to 2002 [16]. The participants from the United States were under diarrhea surveillance when they traveled to Guatemala or Mexico for up to 28 days. Participants were given vials for collection of fecal samples and daily diaries to record the presence or absence of specific gastrointestinal and general symptoms each day during the stay, up to 28 days. The fecal samples were collected from participants when they were asymptomatic (routine samples) as well as when they had diarrhea. Diarrhea was defined as the passage of three or more loose stools during a 24-h period, associated with at least one other symptom, such as abdominal pain, cramps or nausea. More severe episodes were those with either ≥5 loose stools in 24 h, or illness episodes with abdominal cramps, pain or vomiting that interfered with daily activities. The stool samples were cultured in country using MacConkey agar and five *E*. *coli* colonies per sample were sent to JHU in Baltimore to be tested for enterotoxin (LT and ST) using ELISA methods [16,17]. ST was not differentiated as STh and STp. In addition, stool samples were frozen and sent to JHU for long term storage. One hundred and six frozen fecal samples (from both diarrhea cases and asymptomatic patients) and their enterotoxin ELISA results were included in the current study.

### Study 3: Controlled human infection model (CHIM) study at JHU

In an experimental challenge model conducted at JHU, healthy American adult volunteers were challenged with an ETEC strain H10407 in an in-patient unit at JHU [18]. The challenge strain H10407 ETEC serotype O78:H11, produces heat labile toxin (LT) and two forms of heat stable toxin (STh and STp). The stool samples were collected every day following challenge. The stool samples were cultured on MacConkey agar and up to 5 colonies appearing to be *E*. *coli* were tested for agglutination with antiserum to H10407. If at least one of these colonies agglutinated, the sample was considered positive [18].

Thirty available archived frozen stool samples before and after challenge (before antibiotic treatment) that were spotted and dried on Whatman 903 protein saver cards (Millipore, Sigma, MO, USA) in duplicates, along with the culture data were included in the current study. One of the spots, was used for conventional PCR and the other for RLDT.

### Study 4: Surveillance study in India

Stool samples were collected from the acute diarrheal patients under a hospital-based surveillance program by the National Institute of Cholera and Enteric Diseases (NICED), India. The surveillance was conducted on two random days per week by enrolling every fifth diarrheal patient admitted to the Infectious Diseases Hospital in Kolkata, India [19]. The samples were screened for *Shigella spp*. and other enteric pathogens using standard methods at NICED [19]. For detection of *Shigella spp*., stool samples were cultured on Hekton Enteric agar (HEA agar) and *Xylose Lysine Deoxycholate agar* (*XLD agar*), followed by biochemical tests, and serotyped using commercially available antisera (Denka Seiken, Tokyo, Japan) at NICED.

Fifty stool samples, 50μl each were spotted and dried on Whatman 903 protein saver cards in duplicates, were included in the current study. One of the spots was used for conventional PCR and the other for RLDT.

### Quantitative PCR, conventional PCR and RLDT

At JHU, for qPCR, DNA was isolated from 367 frozen stool samples from study 1 and 2. For DNA extraction, 200–300 mg of solid stool or up to 500ul of loose stool was used. DNA was isolated using a bead beater with four 3-mm-diameter solid-glass beads (Sigma-Aldrich) in PBS and subsequently 0.3g of 0.1 mm zirconium beads (BIO-SPEC Inc.) to disrupt cells. The cell slurry was then centrifuged at 16000 *g* for 1 min, the supernatant processed using the Qiagen QIAamp DNA stool extraction kit [20]. QPCR was conducted with the isolated DNA to detect the target genes LT, STh and STp for ETEC and *ipaH* for Shigella using the Step One Plus Real-Time PCR System (Applied Biosystems, CA) with SYBR Green-Based fluorescent dye [21,22]. Each sample was run at a minimum in duplicate, and results were averaged. The 25μl reaction mixtures contained 1X PowerUp SYBR Green Master Mix, 0.2 μM of each primer and 2.5 μL of sample DNA. PCR was carried out for 40 cycles of 95°C for 15s and 60°C for 1 min [21,22]. ETEC strain H10407 and *Shigella flexneri* 2a 2457T were used as positive controls.

All RLDT assays were conducted at JHU, directly from the frozen stool samples using the RLDT kit as described by Chakraborty et al [13]. In short, samples were added to the sample processing tube with lysis buffer followed by heat lysis. The processed samples were then added to the ETEC and Shigella RLDT lyophilized reaction tube (LRT) strips. Each strip consisted of 8 tubes, organized as two reaction tubes each for LT, STh and *ipaH* and one tube for STp. One reaction tube was added as the RLDT inhibitor control [13]. The strips were run for 40 minutes in a real time fluorometer reader (Agdia Inc, IN, USA). The stool from the filter papers were processed as described before [13] using RLDT kit.

Conventional PCR for detection of ETEC and Shigella was done as described before with modifications [15,23]. ETEC strain H10407 and *Shigella flexneri* 2a 2457T were used as the positive controls. *E*. *coli* strain ATCC 25292 was used as negative control. The amplification products were visualized by running in agarose gels.

### Comparison of the assays

The positivity of the samples for ETEC and Shigella were compared using qPCR, RLDT, conventional PCR and culture to determine sensitivity and specificity. The qPCR, PCR from filter paper and RLDT tests were run and interpreted by two lab personnel blinded to each other. “ETEC total” was considered positive when at least one of the ETEC genes, LT, STh or STp was positive. Samples in each study were randomely selected from the culture positives and negatives. The lab personnel performed RLDT, qPCR and PCR, were blinded to the culture results. Sensitivity, and specificity values were expressed as percentages. The sensitivity and specificity of ETEC were also presented as a dot plot using GraphPad Prism 9.3.0. For analysis, the cut off value to determine positives was assigned as the lowest detection limits (LOD) of the RLDT assays [13], 6x10^3^ CFU/g of stool (corresponds to qPCR Cq of 31.03) for Shigella and 9x10^4^ CFU/g of stool for ETEC genes (corresponds to qPCR Cq of 28.2, 28.6 and 30.07 for LT, STh and STp respectively). RLDT and qPCR both can detect ETEC and Shigella with CFU lower than these LOD cut off values. To avoid incorrectly determining some samples to be false positive, samples positive by RLDT with lower CFU (higher Cq) than the LOD cut offs and also positive by qPCR were included as true positives. The results were also analysed with only RLDT LOD as cut off (alternate cut off) which is described in the supporting information (S1 and S2 Tables).

## Results

### Comparison of RLDT to qPCR

In the study 1 and 2 together, out of 367 stool samples screened, 102 (27.8%) were positive for ETEC by RLDT and 101 (27.5%) by qPCR using the assigned Cq cut off values. qPCR was done from the isolated DNA while RLDT was done directly from the stool using RLDT kit. Overall, sensitivity and specificity of RLDT compared to qPCR as gold standard were 99% and 99.2% respectively. The test results by diagnostic methods are presented in Table 1.

**Table 1:** Sensitivity and specificity of ETEC RLDT compared to qPCR.

ETEC qPCR (as the gold standard) vs RLDT							
Targets	Total samples screened	Samples positive by RLDT (%)	Samples positive by qPCR (%)	False positive	False negative	Sensitivity (%)	Specificity (%)
**Overall (includes both study 1 and study 2)**							
**ETEC**	**367**	102 (27.8)	101 (27.5)	2	1	99	99.2
**ETEC Study 1 (Hospital-based surveillance in Bangladesh)**							
ETEC Total	261	62 (23.8)	62 (23.8)	1	1	98.4	99.5
LT	261	36 (13.8)	38 (14.6)	0	2	94.7	100
STh	261	42 (16.1)	42 (16.1)	0	0	100	100
STp	261	15 (5.7)	14 (5.4)	1	0	100	99.6
**ETEC study 2 (overall travelers to Guatemala and Mexico)**							
ETEC Total	106	40 (37.7)	39 (36.8)	1	0	100	98.5
LT	106	23 (21.7)	23 (21.7)	0	0	100	100
STh	106	20 (18.9)	20 (18.9)	0	0	100	100
STp	106	24 (22.6)	24 (22.6)	1	1	95.8	98.8
**ETEC study 2 (travelers with MSD only)**							
ETEC Total	39	25 (64.1)	25 (64.1)	0	0	100	100
LT	39	17 (43.6)	17 (43.6)	0	0	100	100
STh	39	16 (41)	16 (41)	0	0	100	100
STp	39	12 (30.8)	13 (33.3)	0	1	92.3	100
**ETEC study 2 (asymptomatic travelers only)**							
ETEC Total	67	15 (22.4)	14 (20.9)	1	0	100	98.1
LT	67	6 (9)	6 (9)	0	0	100	100
STh	67	4 (6)	4 (6)	0	0	100	100
STp	67	12 (17.9)	11 (16.4)	1	0	100	98.2

In the surveillance study in Bangladesh (study 1), the sensitivity and specificity of RLDT compared to qPCR were 98.4% and 99.5% respectively. The sensitivity and specificity of RLDT for ETEC specific toxin genes ranged from 94.7% to 100% (Table 1).

Among the 106 samples collected from the travelers (study 2), 40 (37.7%) were positive for ETEC by RLDT, 39 (36.8%) by qPCR using the assigned Cq cut offs. The sensitivity and specificity of RLDT for ETEC compared to qPCR were 100% and 98.5% respectively.

Out of 106 traveler’s samples, 39 (36.8%) samples were collected from the volunteers with moderate to severe traveler’s diarrhea (MSD) and 67 (62.6%) samples were collected as routine samples from the volunteers who were either asymptomatic [49 (73.1%)] or with mild diarrhea [18 (26.9%)]. Among the MSD samples, 25 (64.1%) were positive for ETEC by both the RLDT and qPCR with 100% sensitivity and specificity. There was a single false negative for STp by RLDT (Table 2A). Among the 67 asymptomatic or mild diarrhea samples, 15 (22.4%) and 14 (20.9%) were positive for ETEC by RLDT and qPCR respectively. The sensitivity of RLDT was 100% and the specificity was 98.1 to 100% for the ETEC genes.

**Table 2:** Sensitivity and specificity of Shigella RLDT compared to qPCR.

Shigella qPCR (as the gold standard) vs RLDT						
Total samples screened	Samples positive by RLDT (%)	Samples positive by qPCR (%)	False positive	False negative	Sensitivity (%)	Specificity (%)
**Overall (includes both study 1 and study 2)**						
367	9 (2.5%)	9 (2.5%)	0	0	100	100
**Study 1 (Hospital-based surveillance in Bangladesh)**						
261	6 (2.3)	6 (2.3)	0	0	100	100
**Study 2 (overall travelers to Guatemala and Mexico)**						
106	3 (2.8)	3 (2.8)	0	0	100	100
**Study 2 (travelers with MSD only)**						
39	2 (5.1)	2 (5.1)	0	0	100	100
**Study 2 (asymptomatic travelers only)**						
67	1 (1.5)	1 (1.5)	0	0	100	100

The positivity rate for Shigella by both RLDT and qPCR in this sample set (study 1 and 2) was 2.5% (9 positives out of 367 samples screened) with 100% agreement between the two assays (Table 2).

### Comparison of RLDT to culture

The culture followed by colony PCR data for ETEC were available for 230 samples from study 1 (Table 3). The sensitivity and specificity of RLDT for ETEC compared to culture as the gold standard were 96% and 83.4% respectively. The RLDT could detect 34 (16.6%) more ETEC compared to culture. There was one false negative for each LT and STh and 2 for STp by RLDT.

**Table 3:** Sensitivity and specificity of ETEC RLDT compared to culture.

ETEC culture (as the gold standard) vs RLDT							
	Total samples screened	Samples positive by RLDT (%)	Samples positive by culture (%)	False positive	False negative	Sensitivity (%)	Specificity (%)
**ETEC Study 1(Surveillance in Bangladesh) culture followed by PCR vs RLDT**							
ETEC Total	230	58 (25.2)	25 (10.9)	34	1	96	83.4
LT	230	34 (14.8)	9 (3.9)	26	1	88.9	88.2
STh	230	40 (17.4)	19 (8.3)	22	1	94.7	89.6
STp	230	14 (6.1)	7 (3)	9	2	71.4	96
**ETEC study 2 (overall travelers) culture followed by ELISA vs RLDT**							
ETEC Total	106	40 (37.7)	45 (42.5)	3	8	82.2	95.1
LT	106	23 (21.7)	21 (19.8)	6	4	81	92.9
ST	106	36 (34)	37 (34.9)	6	7	81.1	91.3
**ETEC study 2 (travelers with MSD only)**							
ETEC Total	39	25 (64.1)	28 (71.8)	0	3	89.3	100
LT	39	17 (43.6)	14 (35.9)	4	1	92.9	84
ST	39	22 (56.4)	23 (59)	1	2	91.3	93.8
**ETEC study 2 (Asymptomatic travelers)**							
ETEC Total	67	15 (22.4)	17 (25.4)	3	5	70.6	94
LT	67	6 (9)	7 (10.4)	2	3	57.1	96.7
ST	67	14 (20.9)	14 (20.9)	5	5	64.3	90.6

In the traveler’s study (study 2), the sensitivity and specificity of RLDT compared to culture followed by enterotoxin ELISA as gold standard were 82.2% and 95.1% respectively (Table 3). There were 3 false positives and 8 false negatives by RLDT. Among the travelers with MSD, the sensitivity and specificity of RLDT were 89.3% and 100% respectively while among the asymptomatic travelers were 70.4% and 94% respectively. RLDT could detect 3 (6%) more ETEC infections among the asymptomatic individuals which were also positive by qPCR. Culture data were not available for Shigella from study 1 and 2.

### Performance of RLDT from dried stool on filter paper

We evaluated performance of RLDT using dried stool samples on filter paper collected from a CHIM study (study 3) for ETEC and from a surveillance study in India (study 4) for Shigella. Archived frozen stool samples spotted on filter paper were used for RLDT and conventional PCR. Cultures from fresh stools for ETEC were based on agglutination of *E*. *coli* isolates with antiserum to the whole H10407 ETEC bacteria, and standard culture method was used to detect Shigella.

The PCR for ETEC (study 3) was less sensitive compared to RLDT and the later could detect 6 (20%) more ETEC positives which were also positives by culture (Table 4). All the samples positive by PCR were also positive by RLDT. Similar results were seen for detection of Shigella (study 4) (Table 4). Compared to PCR as the gold standard, the sensitivity and specificity of Shigella RLDT were 100% and 90.3% respectively (Table 4). RLDT detected 3 (5%) more Shigella than PCR.

**Table 4:** Sensitivity and specificity of ETEC and Shigella RLDT from the stool on filter paper compared to conventional PCR.

RLDT from stool on filter paper compared with PCR as the gold standard							
Targets	Total samples screened	Samples positive by RLDT (%)	Samples positive by the gold PCR (%)	False positive	False negative	Sensitivity (%)	Specificity (%)
**ETEC CHIM study**							
**ETEC Total** Study 3	30	13 (43.3)	7 (23.3)	6	0	100	73.9
LT	30	13 (43.3)	6 (20)	7	0	100	70.8
STh	30	12 (40)	6 (20)	6	0	100	75
STp	30	13 (43.3)	4 (13.3)	9	0	100	65.4
**Hospital-based surveillance in India**							
**Shigella** Study 4	59	31 (52.54)	28 (48)	3	0	100	90.3

The sensitivity and specificity of ETEC RLDT (study 3) from filter paper compared to culture as the gold standard were 86.7% and 100% respectively with 2 false negatives (Table 5). The false negatives were also negative by PCR. When compared to the culture as the gold standard, RLDT could detect 11(19%) more Shigella resulting in 100% sensitivity and 71.8% specificity (Table 5).

**Table 5:** Sensitivity and specificity of ETEC and Shigella RLDT from the stool on filter paper compared to culture.

RLDT from stool on filter paper compared with culture as the gold standard							
Targets	Total samples screened	Samples positive by RLDT (%)	Samples positive by culture (%)	False positive	False negative	Sensitivity (%)	Specificity (%)
**ETEC CHIM study**							
ETEC Study 3	30	13 (43.3)	15 (50)	0	2	86.7	100
**Hospital-based surveillance in India**							
Shigella Study 4	59	31 (52.54)	20 (34)	11	0	100	71.8

**Fig 1:**
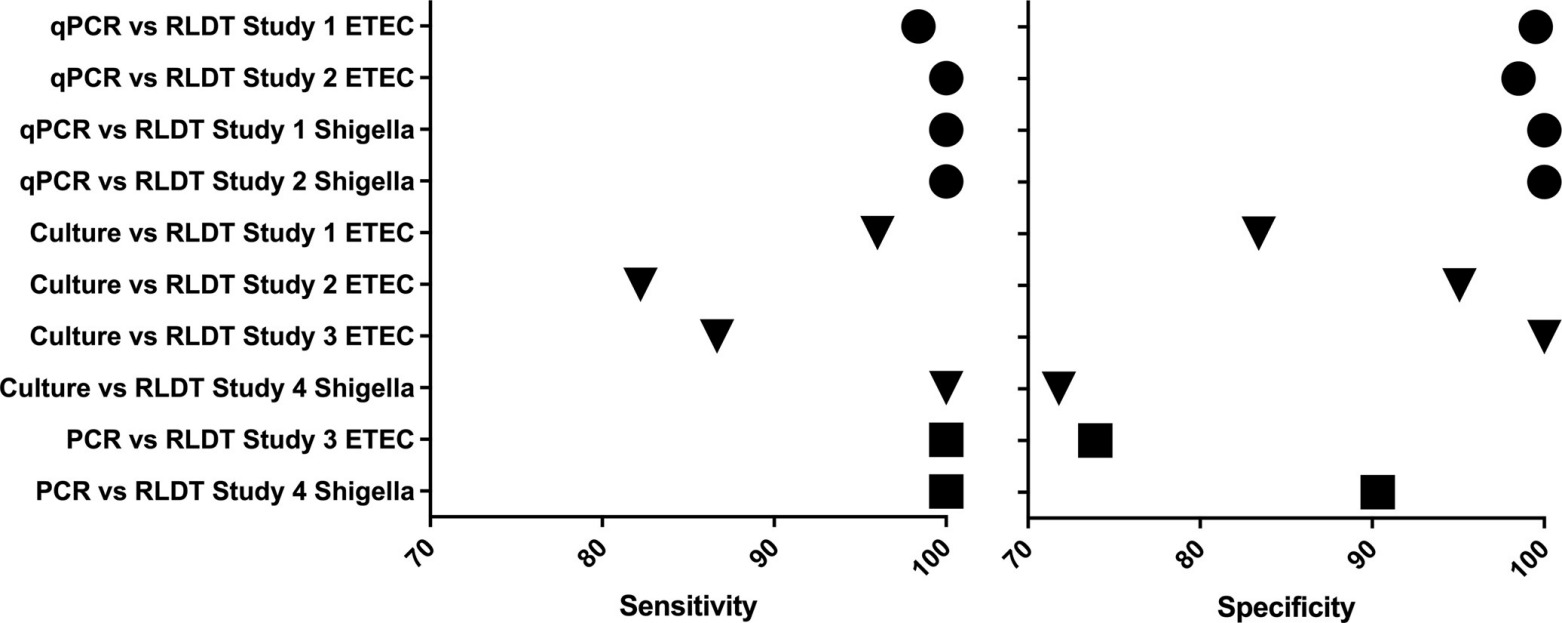
Dot plot of sensitivity (left) and specificity (right) of ETEC and Shigella RLDT compared to qPCR (circle); culture (triangle) and PCR (square).

The sensitivity and specificity of ETEC and Shigella RLDT compared to other assays from all the studies are summarized as a dot plot in Fig 1.

## Discussion

In this study, we evaluated the diagnostic performance of RLDT for ETEC and Shigella compared to qPCR, conventional PCR and culture. Overall, RLDT had excellent sensitivity (99%) and specificity (99.2%) for ETEC and Shigella (100%) compared to qPCR. The sensitivity and specificity of RLDT in detection of individual toxin genes of ETEC ranged from 92.3 to 100% and 98.1 to 100% respectively. We tested RLDT in the stool samples obtained from two different population settings, from the patients with acute diarrhea seeking care at the hospitals in Bangladesh and India and from the travelers visiting to Guatemala and Mexico where infection with enteric pathogens is common. These two sample sets are different in nature as co-pathogens and previous immunity from repeated infections are expected among the patients in the endemic countries whereas travelers to these countries might have limited or no previous exposures to the enteric pathogens. The sensitivity and specificity of RLDT were similar in theses samples. The diagnostic performance of RLDT compared to qPCR was also similar in the samples from the volunteers with diarrhea and in those who were asymptomatic. As reported in previous studies [24,25] the sensitivity of culture was lower compared to RLDT and qPCR. Similar to our study 4, secondary analysis of the Global Enteric Multicenter Study (GEMS) reported that culture missed half of Shigella-attributed MSD cases in that study and culture also missed over half of the Shigella-associated deaths [26]. The lower sensitivity of culture could be due to *E*. *coli* being a commensal organism and a selective media for either ETEC or Shigella is not available. Therefore, the sensitivity of culture depends on the number of isolates tested, whereas the RLDT and qPCR assay examined stool in its entirety. However, there were few samples positive by enterotoxin ELISA, which were negative by both RLDT and qPCR.

It should be noted that, the frozen stool samples which were used in this study for RLDT and qPCR or spotted on filter paper for RLDT and conventional PCR were stored for minimum of 4 years (study 1) to maximum of 21 years (study 2) before used in this study, while the cultures were done from fresh stool samples in real time during those studies. It is noteworthy that, despite these differences, the concordance between RLDT and qPCR as well as RLDT and culture were very high.

This study also determined that RLDT could be performed on dried stool samples on filter paper, exhibiting high detection sensitivity for both enteric pathogens. Thus, when RLDT cannot be performed on site, such as during humanitarian emergencies or in very remote areas, the stool samples can be spotted on filter paper, dried and sent to a lab where the RLDT can be carried out.

As described previously, although, the nucleic acid tests from stool are more sensitive than culture, culture-based detection methods (based on testing of colonies) remain crucial for downstream applications like whole genome sequencing, antibiotic resistance monitoring, serotyping, colonization factors (CFs) typing, serotype and CF-specific immunity in vaccine trials.

Since RLDT is rapid (<1hour), this test can be used also as a screening test so only stool samples that are positive for ETEC or Shigella by RLDT need to be cultured to isolate colonies and this could be done on the same day. This would save time, labor and resources that would have been required to culture all the samples. In addition, for ETEC detection, since following RLDT, only positive stool samples could be selected to culture, the number of colonies tested per sample could be increased to maximize the probability of detecting ETEC positive isolates. The RLDT may increase the sensitivity of culture allowing for reduced sample size required in the phase III trials of ETEC and Shigella vaccines if serotype and CFs immunity and efficacy are determined by culture. As an example of the potential improved efficiency when using RLDT, during the study of travelers’ diarrhea in Guatemala and Mexico, 2355 fecal specimens were collected which required isolation and testing of more than 11,000 isolates. Very few of these fecal specimens were positive for ETEC (estimated to be about 5%). If RLDT had been used, this would have significantly reduced the number of specimens requiring subsequent testing.

If a large field trial of an ETEC or shigella vaccine was to be conducted, the number of samples to be tested would be hundreds of times higher and would not be feasible without a screening procedure like RLDT.

Another significant advantage of RLDT, since simple and rapid, could be used as a point of care diagnostic test for clinical decision making. In the era of increasing antibiotic resistance, informed decision in appropriate use of antibiotics is crucial.

With the enteric pathogens TaqMan array used in the reanalysis of the GEMS studies [24], the diarrhea associated quantity for Shigella (*ipaH* gene) was found to be a Cq value of 33.1 (corresponding to 2.1x10^6^ CFU/g of stool) and for ETEC-STh, a Cq value of 26.2 (corresponding to 2.0x10^7^ CFU/g of stool). The relation of Cq value to CFU/g of stool varies by the qPCR assay chemistry, master mix, assay protocol and the equipment used. The LOD for Shigella and ETEC in the RLDT assay were approximately 2 logs lower (13) than the diarrhea associated quantity defined in the above study. Based on the results of this study, it can be concluded that RLDT is able to identify the clinically relevant symptomatic and asymptomatic ETEC and Shigella infections and the sensitivity of RLDT is sufficient to better determine disease burden estimates for these two pathogens. It should also be noted that, as described before (13), if required, the RLDT reader could be programmed with more stringent cut offs to determine ETEC and Shigella positive/negative samples (13) which were reported to be highly associated with ETEC and Shigella MSD [24,26].

There are limitations in this study. The three assays compared here, used various starting materials since the different assays required different sample preparation methods. RLDT was done directly from the stool with minimum sample treatment; qPCR was done from purified DNA; PCR was done from filter papers; culture was done by either culture followed by PCR or enterotoxin ELISA with ETEC isolates. Therefore, the sensitivity of these assays depends not only on the amplification technology but also on the starting material. In addition, while qPCR is quantitative, RLDT is semi-quantitative, PCR and culture are qualitative assays.

In conclusion, RLDT exhibited excellent and sufficient sensitivity and specificity for the detection of clinically relevant levels of ETEC and Shigella infection. Based on the results reported here, RLDT warrants broader application and evaluation as a culture-independent simple and rapid diagnostic test for these bacteria in resource poor settings where such simple assays are greatly needed [26]. In this regard, RLDT assay is currently undergoing validation and implementation in endemic country study sites to further ascertain its potential to determine the impact of ETEC and Shigella among infants and children living in high-risk areas for enteric infections. In addition, the RLDT assay is also be applied in support of ongoing Phase 2b and 3 trials designed to evaluate the protective efficacy of ETEC and Shigella vaccines.

## Supporting information

S1 Table Sensitivity and specificity of ETEC RLDT comparing with qPCR using alternate cut off. (DOCX)

S2 Table Sensitivity and specificity of Shigella RLDT comparing with qPCR using alternate cut off. (DOCX)
