# Supplementary material for: Evaluation of a simple, rapid and field-adapted diagnostic assay for enterotoxigenic E. coli and Shigella
Source: PLoS Negl Trop Dis. 2022 Feb 7;16(2):e0010192. doi: 10.1371/journal.pntd.0010192 (PMC8853640; doi:10.1371/journal.pntd.0010192)
Supplement: S1 Table — (DOCX) [file pntd.0010192.s001.docx]

**S1 Table. Sensitivity and specificity of ETEC RLDT comparing with qPCR using alternate cut off.**

| **Targets** | **Total samples screened** | **Samples**  **positive by RLDT**  **(%)** | **Samples positive by the gold standard**  **(%)** | **False positive** | **False negative** | **Sensitivity**  **(%)** | **Specificity**  **(%)** |
| --- | --- | --- | --- | --- | --- | --- | --- |
| **Overall (includes both study 1 and study 2)** | | | | | | | |
| ETEC | 367 | 102 (27.8%) | 87 (23.7%) | 16 (5.7%) | 1 (1.1%) | 98.9 | 94.3 |
| **ETEC Study 1 (Surveillance in Bangladesh)** | | | | | | | |
| ETEC  Total | 261 | 62 (23.8) | 51 (19.5) | 12 | 1 | 98 | 94.3 |
| LT | 261 | 36 (13.8) | 32 (12.3) | 6 | 2 | 93.8 | 97.4 |
| STh | 261 | 42 (16.1) | 34 (13) | 8 | 0 | 100 | 96.5 |
| STp | 261 | 15 (5.7) | 7 (2.7) | 8 | 0 | 100 | 96.9 |
| **ETEC study 2 (travelers study)** | | | | | | | |
| ETEC  Total | 106 | 40 (37.7) | 36 (34) | 4 | 0 | 100 | 94.3 |
| LT | 106 | 23 (21.7) | 23 (21.7) | 0 | 0 | 100 | 100 |
| STh | 106 | 20 (18.9) | 19 (17.9) | 1 | 0 | 100 | 98.9 |
| STp | 106 | 24 (22.6) | 21 (19.8) | 4 | 1 | 95.2 | 95.3 |
