# Supplementary material for: Evaluation of a simple, rapid and field-adapted diagnostic assay for enterotoxigenic E. coli and Shigella
Source: PLoS Negl Trop Dis. 2022 Feb 7;16(2):e0010192. doi: 10.1371/journal.pntd.0010192 (PMC8853640; doi:10.1371/journal.pntd.0010192)
Supplement: S2 Table — (DOCX) [file pntd.0010192.s002.docx]

**S2 Table. Sensitivity and specificity of Shigella RLDT comparing with qPCR using alternate cut off.**

| **Total samples screened** | **Samples positive by RLDT**  **(%)** | **Samples**  **positive by the gold standard**  **(%)** | **False positive** | **False negative** | **Sensitivity**  **(%)** | **Specificity**  **(%)** |
| --- | --- | --- | --- | --- | --- | --- |
| **Overall (includes both study 1 and study 2)** | | | | | | |
| 367 | 9 (2.5) | 9 (2.5) | 0 | 0 | 100 | 100 |
| **Study 1 (Surveillance in Bangladesh)** | | | | | | |
| 261 | 6 (2.3%) | 6 (2.3%) | 0 | 0 | 100 | 100 |
| **Study 2 (overall travelers)** | | | | | | |
| 106 | 3 (2.8) | 3 (2.8) | 0 | 0 | 100 | 100 |
